# Computed tomographic evaluation of myocardial ischemia

**DOI:** 10.1007/s11604-020-00922-8

**Published:** 2020-02-05

**Authors:** Yuki Tanabe, Akira Kurata, Takuya Matsuda, Kazuki Yoshida, Dhiraj Baruah, Teruhito Kido, Teruhito Mochizuki, Prabhakar Rajiah

**Affiliations:** 1 Department of Radiology, Ehime University Graduate School of Medicine, Shitsukawa, Toon, Ehime 791-0295 Japan; 2 Department of Radiology, Medical College of Wisconsin, Milwaukee, WI USA; 3 Department of Radiology, I.M. Sechenov First Moscow State Medical University, Bol’shaya Pirogovskaya Ulitsa, Moscow, Russia; 4 Department of Radiology, Mayo Clinic, Rochester, MN USA

**Keywords:** Computed tomography, Coronary artery disease, Myocardial ischemia, Myocardial perfusion, Fractional flow reserve

## Abstract

Myocardial ischemia is caused by a mismatch between myocardial oxygen consumption and oxygen delivery in coronary artery disease (CAD). Stratification and decision-making based on ischemia improves the prognosis in patients with CAD. Non-invasive tests used to evaluate myocardial ischemia include stress electrocardiography, echocardiography, single-photon emission computed tomography, and magnetic resonance imaging. Invasive fractional flow reserve is considered the reference standard for assessment of the hemodynamic significance of CAD. Computed tomography (CT) angiography has emerged as a first-line imaging modality for evaluation of CAD, particularly in the population at low to intermediate risk, because of its high negative predictive value; however, CT angiography does not provide information on the hemodynamic significance of stenosis, which lowers its specificity. Emerging techniques, e.g., CT perfusion and CT-fractional flow reserve, help to address this limitation of CT, by determining the hemodynamic significance of coronary artery stenosis. CT perfusion involves acquisition during the first pass of contrast medium through the myocardium following pharmacological stress. CT-fractional flow reserve uses computational fluid dynamics to model coronary flow, pressure, and resistance. In this article, we review these two functional CT techniques in the evaluation of myocardial ischemia, including their principles, technology, advantages, limitations, pitfalls, and the current evidence.

**Electronic supplementary material:**

The online version of this article (10.1007/s11604-020-00922-8) contains supplementary material, which is available to authorized users.

## Introduction

**Fig. 1:**
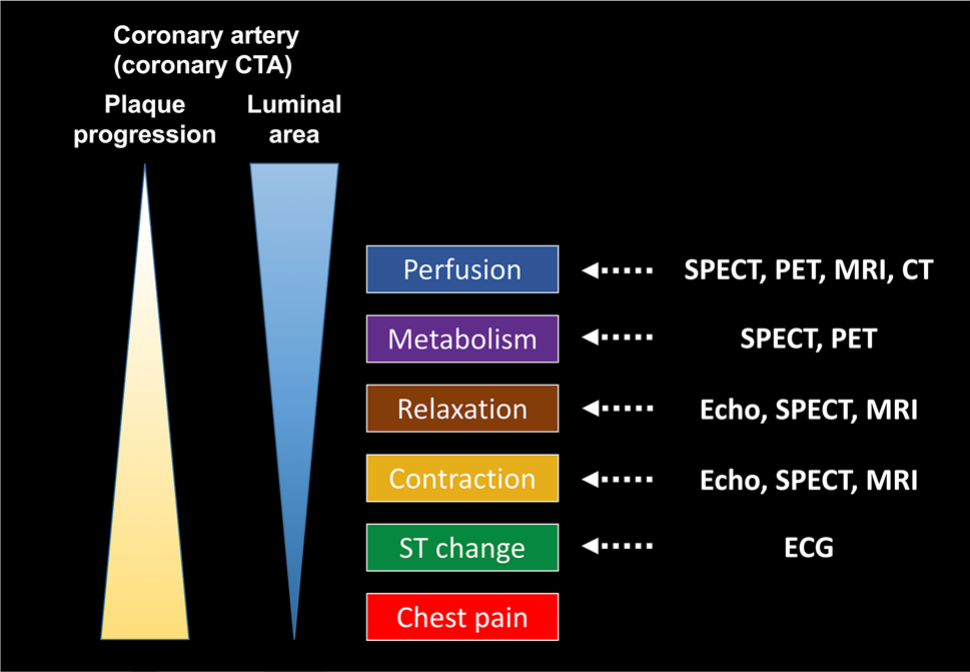
Illustration showing the progressive pathological conditions in the myocardial ischemic cascade. Coronary artery atherosclerosis progresses and leads to myocardial hypoperfusion because of plaque progression and luminal stenosis. Myocardial hypoperfusion is followed by metabolic abnormalities, diastolic dysfunction, systolic dysfunction, and ECG abnormalities, culminating in chest pain. The right column shows the modalities that can be used to detect abnormalities at each step of the cascade. *CT* computed tomography, *CTA* computed tomography angiography, *ECG* electrocardiogram, *SPECT* single-photon emission computed tomography, *PET* positron emission tomography, *MRI* magnetic resonance imaging

Coronary artery atherosclerosis progresses asymptomatically in the early stage and leads to luminal stenosis and myocardial ischemia [1]. The ischemic cascade illustrates the progressive pathological conditions that develop from hemodynamically significant stenosis, evolving from subclinical to clinical stages (Fig. 1) [2–4]. Decreased perfusion leads to metabolic changes, followed by diastolic and then systolic dysfunction, electrocardiographic (ECG) changes, and anginal chest pain [3]. Blood flow and contractile function in myocardial ischemia can be improved by medical therapy or revascularization procedures such as percutaneous coronary intervention (PCI) or coronary artery bypass grafting (CABG). Large multicenter trials have demonstrated improvement in the prognosis of coronary artery disease (CAD) by decision-making according to myocardial ischemia shown on stress testing [5, 6]. Therefore, current international guidelines require proof of myocardial ischemia before a revascularization procedure [7, 8]. This can be achieved by multiple noninvasive and invasive tests, each with advantages and disadvantages (Table 1).

**Table 1:** Advantages and disadvantages of different tests used in the evaluation of myocardial ischemia

Modality	Advantages	Disadvantages
Electrocardiogram	Availability	Depends on patient activity and cooperation with the test
	Cost-effectiveness	Low sensitivity for ischemia
	Multipurpose use for ischemia, exercise tolerance, and therapeutic effect	Diagnostic difficulty on a per-vessel basis
Echocardiogram	High temporal resolution	Two-dimensional cross-sectional images
	Differential assessment of diastolic and systolic dysfunction	Depends on operator experience, image quality, and limited acoustic window
	Myocardial strain imaging	
CT	High spatial resolution	Radiation exposure
	Assessment of coronary artery stenosis and plaque	Limited temporal resolution
	CT perfusion (ischemia)	Intolerance to irregular heartbeat
	Late iodine enhancement (infarction)	Contrast contamination by preceding protocol
	CT-FFR (computational lesion-specific assessment of myocardial ischemia)	Contrast-related complications (kidney, allergy, and chronic lung disease)
Nuclear imaging (SPECT, PET)	Abundant evidence	Less spatial resolution
	Tracer selection by purpose	No information on coronary anatomy
	ECG-gated scan (perfusion and wall motion)	Radiation dose
	Image fusion	Cost and throughput
	Myocardial viability	
MRI	High spatial resolution	Contraindications (metallic device, claustrophobia)
	High contrast resolution	Contrast-related complications (brain deposition, nephrogenic systemic fibrosis)
	No ionizing radiation exposure	Throughput (long examination time)
	Differentiation of ischemia and infarction	Susceptible to arrhythmia
Invasive FFR	Lesion-specific assessment of myocardial ischemia	Invasive procedure and risk of complications
	Established evidence for decision-making and prognosis	Complexity for repeat pharmacological stress

*CT* computed tomography, *ECG* electrocardiogram, *FFR* fractional flow reserve, *MRI* magnetic resonance imaging, *PET* positron emission tomography, *SPECT* single-photon emission computed tomography

Noninvasive tests include exercise ECG, echocardiography, nuclear imaging, stress magnetic resonance imaging (MRI), and computed tomography (CT) and evaluate different stages along the ischemic cascade. The choice of test to symptomatic patients is determined by the pre-test probability of CAD and the inspection characteristics including invasiveness, cost, and accessibility. Cardiac CT is widely used as coronary CT angiography (CTA), allowing direct visualization of coronary artery stenosis, and plays an important role in the diagnostic management of CAD [7]. The other non-invasive tests can evaluate myocardial ischemia, but leave some difficulties on the detection of culprit lesion causing ischemia. The invasive fractional flow reserve (FFR) can evaluate the lesion-specific ischemia compared to the morphological stenosis assessed by invasive coronary angiography (ICA) under the conditions of maximum hyperemia [9, 10]. The FFR correlates poorly with stenosis on ICA, and many stenoses that are significant on ICA (≥ 50% stenosis) do not have a significant low FFR (≤ 0.8) [5, 10]. The clinical outcome has been shown to be better when revascularization decisions are based on the FFR than on visual estimation of the severity on ICA [5, 11–13].

Japan has the most computed tomography (CT) scanners among those of the Organization for Economic Co-operation and Development (OECD) countries [14]. In Japan, cardiac CT is more common than other modalities (MRI, SPECT, PET), and the number of examinations is also increasing because of the high accessibility and diagnostic performance for detecting coronary artery stenosis [15]. Recent CT technological developments such as high-speed gantry rotation, wide-detector, and iterative reconstruction have increased the value of coronary CTA. Moreover, they allow us to assess myocardial ischemia using stress CTP or CT-derived FFR estimation based on computational flow dynamics. In this review, we review the clinical usefulness of CT-based diagnostic tools in assessing myocardial ischemia from the methodology to advantages and clinical limitations.

## Coronary CTA in CAD

Coronary CTA has high diagnostic performance for prediction of significant coronary stenosis on ICA (i.e., ≥ 50%) with sensitivity of 89%, specificity of 96%, positive predictive value (PPV) of 78%, and negative predictive value (NPV) of 98% on per-segment basis [16]. The high NPV makes coronary CTA valuable for excluding CAD in the population at low or intermediate risk and acting as a gatekeeper for ICA. Up to 63% of elective ICA procedures show non-obstructive disease despite previous functional tests, resulting in suboptimal resource utilization [17]. However, CTA is limited in terms of revealing the hemodynamic significance of a stenosis, given that there is no correlation between the severity of stenosis on CTA and the functional consequences. Only 49% of significant coronary stenosis on CTA (≥ 50% reduction in diameter) correlates with the gold standard of invasive FFR (< 0.75) [18]. Possible causes for this poor correlation include visual overestimation of luminal stenosis and classification of lesions with heavy calcification or motion as “positive” [19]. As a result of the poor specificity, patients with ≥ 50% stenosis on CTA are recommended to undergo further investigation with a functional test to estimate the hemodynamic significance of the stenosis [20].

## CT perfusion

### Principle of CTP

**Fig. 2:**
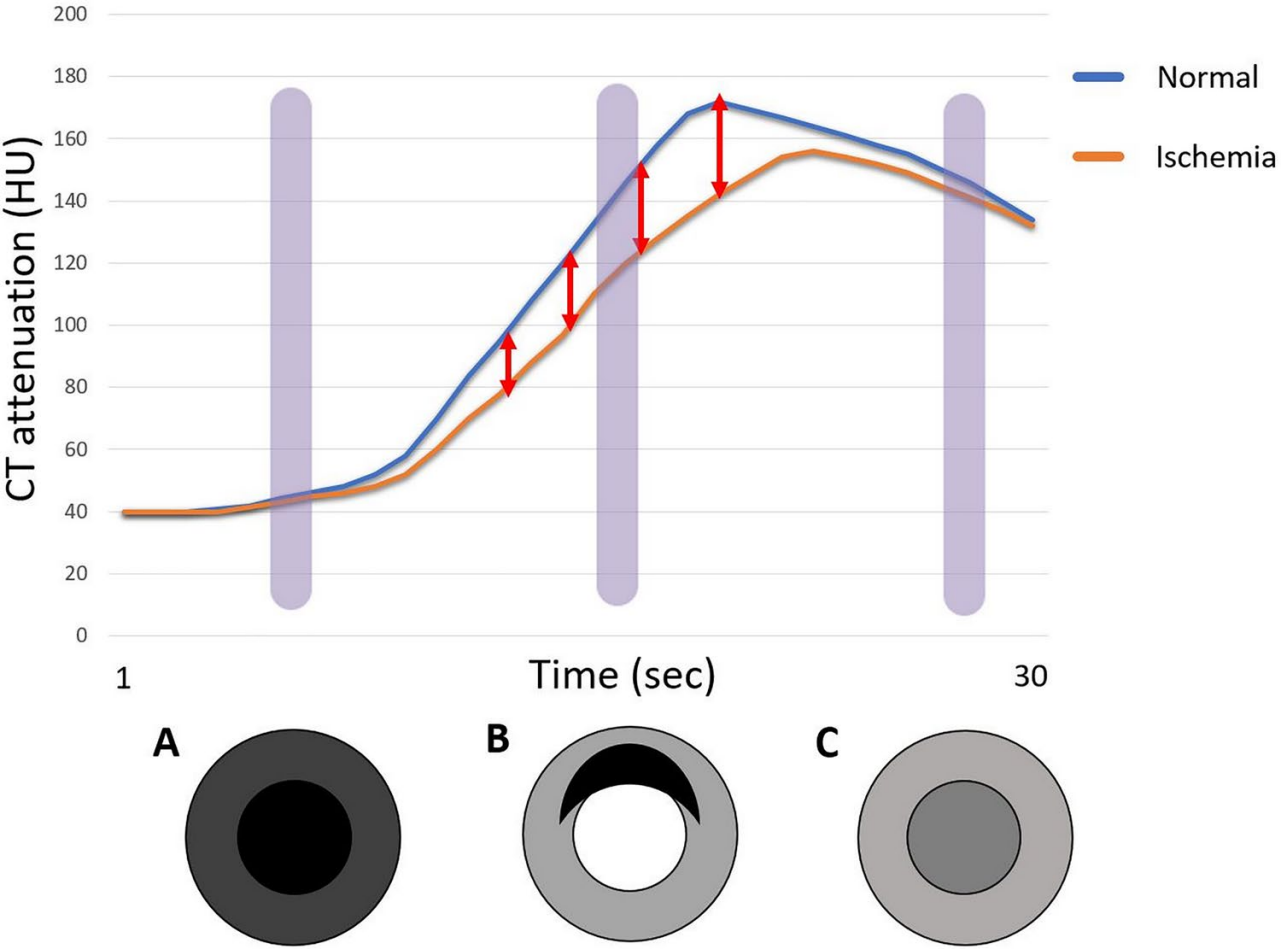
Time attenuation curves for normal myocardium (blue) and ischemic myocardium (orange). At the optimal scan time (B), stress CT perfusion demonstrates a clear distinction between a normal and ischemic myocardium because of large differences in attenuation between normal and abnormal myocardium. Stress CT perfusion cannot distinguish normal from ischemic myocardium if the timing is too early (A) or too late (C). *CT* computed tomography, *HU* Hounsfield unit

CTP evaluates the first pass of contrast medium through the myocardium at rest and during pharmacological stress. CTP emphasizes the differences in perfusion between normal and ischemic myocardium (Fig. 2) [21]. Myocardial perfusion is a complex process involving the coronary arteries and microvasculature. Coronary artery stenosis decreases the myocardial blood flow and perfusion pressure whereas the microvessels dilate to decrease resistance and maintain resting myocardial perfusion even at 80% luminal stenosis [22]. Autoregulation is limited at rest when the stenosis is severe and during stress even in the earlier stages due to higher myocardial oxygen consumption in these states [22].

### Stress agents for CTP

Hyperemia is induced by intravenous administration of a vasodilator stress agent, such as adenosine, adenosine triphosphate (ATP), dipyridamole, or regadenoson. Adenosine binds to the A2A adenosine receptor, leading to coronary vasodilation. It is injected at a rate of 0.14 mg/kg/min for 6 min and has a rapid onset of action and a short half-life of 1–5 s. ATP is metabolized to adenosine and acts in the same way as adenosine. It is injected intravenously at a rate of 0.16 mg/kg/min for 5 min and has a half-life of ≤ 20 s. Both these agents are cleared by cellular mechanisms. Dipyridamole acts indirectly by preventing intracellular reuptake and transport of adenosine by endothelial cells and by increasing endogenous adenosine levels. It is injected intravenously at a rate of 0.14 mg/kg/min for 4 min, has a half-life of 30–45 min, and is cleared by liver. Adenosine, ATP, and dipyridamole are non-selective adenosine receptor agonists and cause flushing, headache, lightheadedness, and chest discomfort, and these side effects are naturally treated due to the very short half-life of adenosine or ATP. Regarding dipyridamole, aminophylline (50–250 mg intravenously at least 1 min after the tracer injection) is used if necessary. Regadenoson is a selective A2A agonist that has a lower risk of side effects. It is injected as a bolus of 0.4 mg over 10 s and is followed by a saline flush; it has a half-life of 33–108 s and is cleared by the kidney [23–25]. Patients should be instructed to avoid consuming caffeine-containing products for ≥ 24 h prior to the scheduled test to prevent the interference with the coronary vasodilatory effects.

### Acquisition techniques of CTP

#### Static CTP imaging

**Fig. 3:**
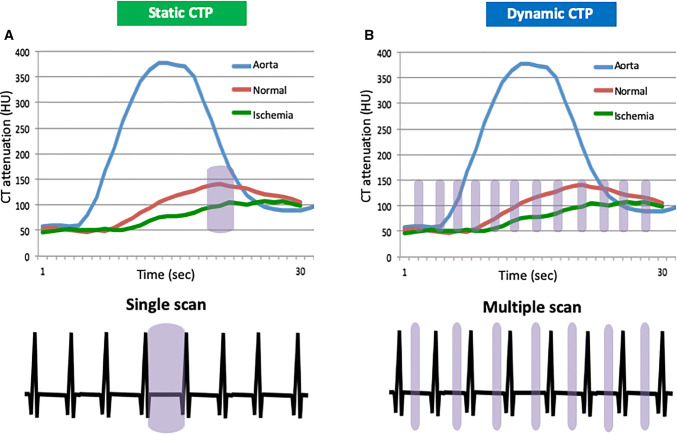
Static and dynamic CTP techniques. **a** Static CTP data are acquired during a single phase of first pass of contrast in the myocardium. **b** Dynamic CTP data are acquired at multiple phases of first pass of contrast in the myocardium. Blue box means scan duration. *CTP* computed tomography perfusion, *HU* Hounsfield unit

Static CTP refers to the single sampling of perfusion during the first pass of iodinated contrast in the myocardium (Fig. 3a) [26]. Scan timing is critical for detecting the perfusion abnormality when this technique is used. A bolus-tracking or timing-bolus technique can aid accurate calculation of the scan time. The optimal scan time is approximately 2–10 s from the time of peak enhancement in the ascending aorta [27, 28]. Scan timing is affected by many factors, including cardiac output, injection rate, and the severity of the perfusion abnormality. Therefore, prospective estimation of the optimal scan timing is challenging. In static CTP, stress and rest CTP images are acquired with either prospective ECG triggering or retrospective ECG gating during a single breath-hold. Acquisitions with retrospective ECG gating provide additional information on wall motion during the cardiac cycle. As a result of improved spatial and temporal resolution of CT scanner, there is now potential to simultaneously evaluate both myocardial and coronary artery perfusion by stress static CTP alone using a low effective radiation dose (2.5 ± 1.1 mSv) [29]*.*

#### Dynamic CTP imaging

Dynamic CTP imaging refers to the acquisition of multiple samples during first pass of iodinated contrast in the myocardium (Fig. 3b). Unlike static CTP, the diagnostic performance of this technique is independent of the optimal scan timing and it also allows quantification of myocardial perfusion [30]. The prospective ECG-triggered acquisition in the systolic phase (40% R–R interval) is advantageous because this phase is less affected by motion artifact and the hypoenhancement is more visible than in the diastolic phase [31]. Motion in the target phase can be reduced by motion correction algorithms that analyze per-vessel and per-segment motion at the voxel level using information from adjacent cardiac phases within a single cardiac cycle [32]. High temporal resolution and wide detector coverage are desirable to obtain perfusion data for the whole heart. Dual-source CT has high temporal resolution (up to 66 ms), which allows acquisition of motion-free images, even at high heart rates. Coronary CTA derived from dynamic CTP imaging by third-generation dual-source CT is useful for diagnosis of coronary artery stenosis [33]. The ECG-triggered axial shuttle mode available on dual-source CT allows acquisition of a dynamic CTP dataset for the whole heart en bloc using rapid movement between two table positions. Although the dynamic acquisitions are performed every third or more heartbeat, dynamic CTP with dual-source CT has been reported to have good diagnostic performance for detecting a myocardial perfusion abnormality [34]. Wide-detector CT (e.g., 256 or 320 slice detectors, with 8 cm/16 cm z-coverage) enables acquisition of data in consecutive heartbeats, providing whole-heart perfusion without temporal gaps [30, 35]. Dynamic CTP imaging by wide-detector CT can accurately quantify myocardial perfusion and is comparable to positron emission tomography (PET) with ^15^O-labelled water, which is the gold standard tracer because it uses a freely diffusible tracer with a 100% extraction fraction even at high blood flow [36]. The radiation dose required for dynamic CTP is substantially higher than that for static CTP [37]. However, advances in CT scanners, the scan protocol, and reconstruction techniques (e.g., low tube voltage, iterative reconstruction) can reduce radiation exposure (< 4 mSv) without impairing image quality [38–40].

### CTP scan protocols

#### Stress CTP-first, rest CTP-first, or stress CTP-only

There are two types of CTP protocols, namely stress-first and rest-first (Fig. 5). Rest CTP also serves as CTA. A gap of at least 10–20 min is allowed between stress and rest CTP to reduce the effects of the medication(s) used in the preceding scan [21]. The advantages of stress-first protocol are the higher sensitivity for detecting ischemia than rest-first protocol and optimized CTA at the second acquisition because of the ability to administer medications (e.g., β-blockers or nitrates) necessary for good image quality without interfering with assessment of stress perfusion. The advantages of rest-first protocol are the higher sensitivity for detecting myocardial infarction than stress-first protocol and the ability to abandon stress CTP if there are no significant lesions in the preceding CTA [25]. However, in the rest-first protocol, contrast contamination from the rest acquisition may hamper the diagnostic performance of the subsequent stress CTP. A rest-first protocol is preferable for patients with a low to intermediate pre-test probability of CAD while a stress-first protocol is preferable for those with a high pre-test probability of CAD, extensive coronary artery calcifications, or a known history of CAD/PCI or myocardial infarction [23]. Regarding dynamic CTP imaging, a stress-only CTP protocol is often adopted because of the limit of radiation exposure. Although a stress-only CTP protocol cannot yield a quantification of coronary flow reserve (CFR), the diagnostic performance is still high thanks to the routine quantitative technique as mentioned in detail later [34, 35, 41].

### Optional scan protocol

#### Dual energy CTP

**Fig. 4:**
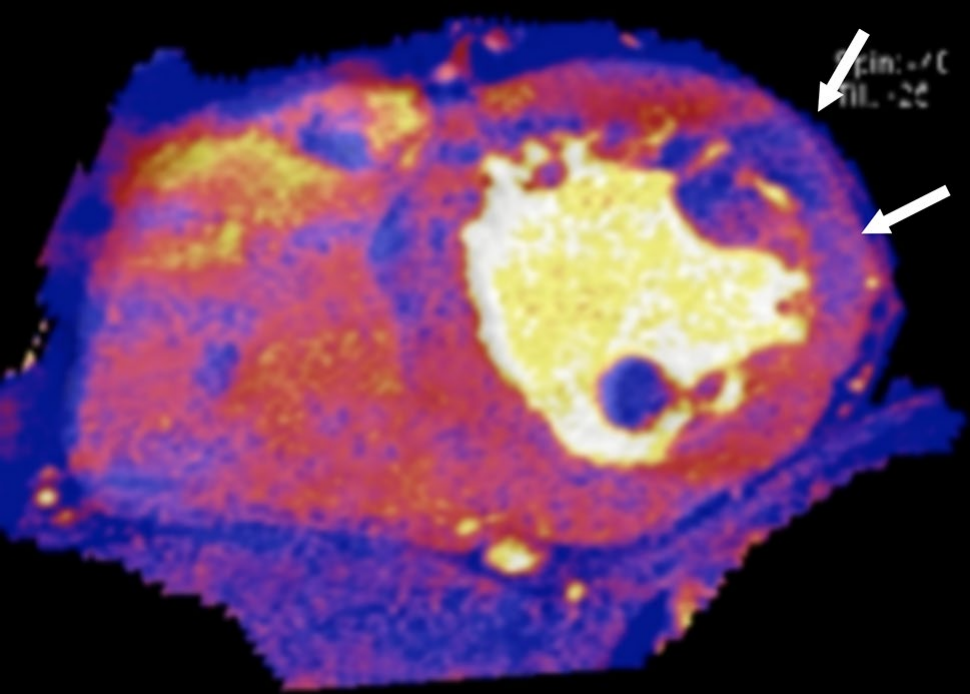
A dual-energy CT scan overlaid with iodine in the short-axis plane obtained from a static stress myocardial CTP scan showing a perfusion defect in the mid anterolateral septum (arrow). *CT* computed tomography, *CTP* computed tomography perfusion

Dual-energy CT (DECT) refers to acquisition of CT data at two different energy levels. DECT allows for characterization of materials with similar attenuation coefficients but different atomic numbers because the tissues show different attenuation properties at different energy levels [42]. Commonly used DECT techniques include dual-source, rapid kVp switching, and dual-layer detector technologies [43]. DECT allows generation of multiple additional images, including iodine maps (Fig. 4), and virtual monoenergetic images (VMIs). Iodine maps highlight pixels containing iodine and can discriminate normal, ischemic, and infarcted myocardium on stress CTP [44] (Fig. 4). Iodine maps have better diagnostic performance than conventional images [43] and also provide quantitative information [45]. VMIs mimic images obtained at a single energy level and can be generated from 40 to 200 keV. Low-energy VMIs (i.e., less than 70 keV) show higher attenuation of iodine because of approximation with its K-edge. Hence, perfusion defects are more conspicuous due to the enhanced contrast between normal and ischemic myocardium [46]. High-energy VMI is beneficial for reducing beam-hardening artifact [47]. Use of VMI is a balance between image contrast and these artifacts.

#### Late iodine enhancement CT

**Fig. 5:**
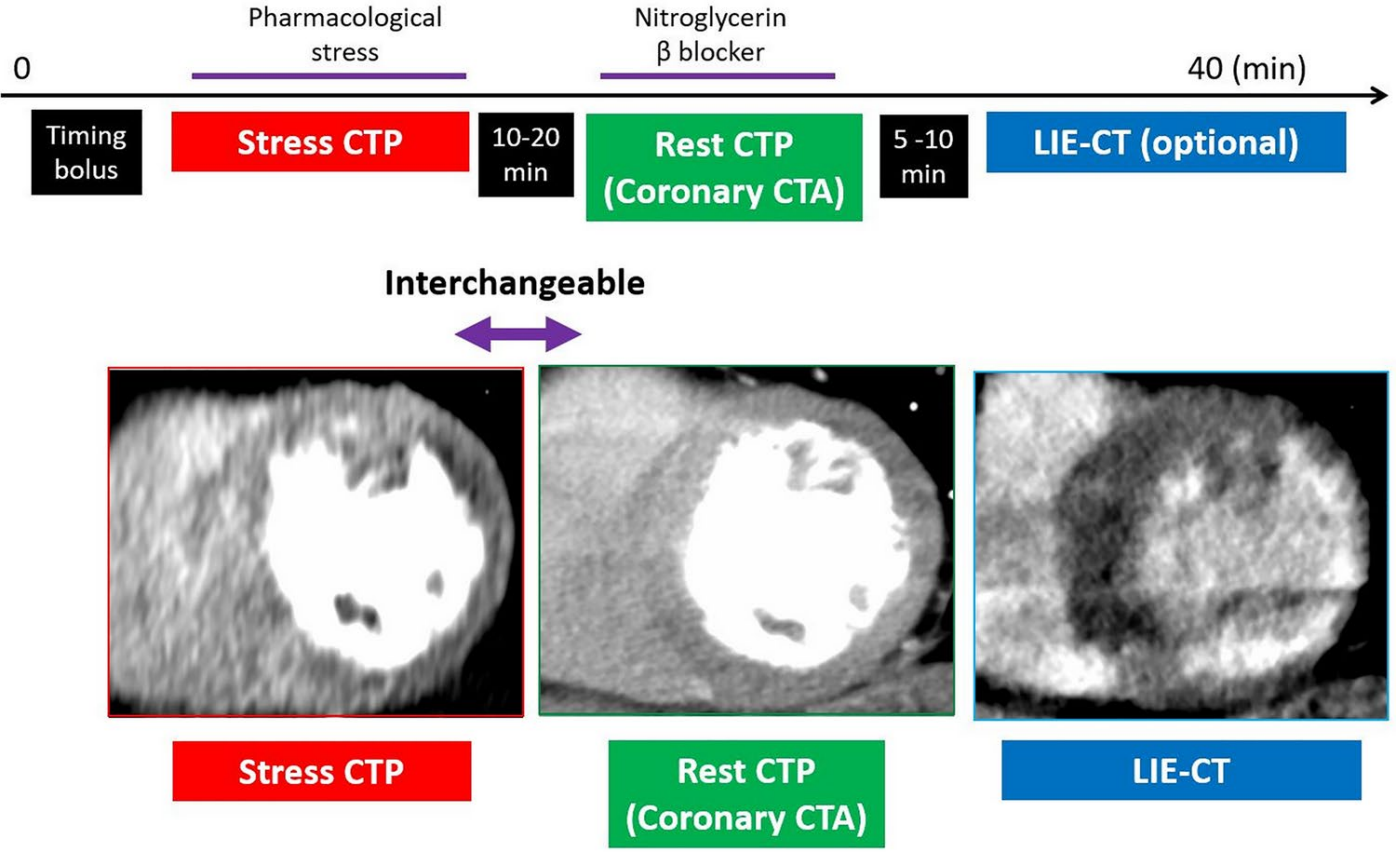
Comprehensive CTP protocol. In the stress-first protocol, CTP is first acquired with pharmacological stress. The rest CTP is acquired 10–20 min later and also serves as coronary CTA. Nitroglycerin and β-blockers are administered during this phase to obtain high-quality coronary CTA images. LIE-CT scans can be obtained 5–10 min following the second CTP scan. In the rest-first protocol, rest images are acquired first and stress images are acquired later. *CTA* computed tomography angiography, *CTP* computed tomography perfusion, *LIE-CT* late iodine enhancement computed tomography

Late iodine enhancement CT (LIE-CT) refers to a delayed phase acquisition following CTP/CTA, which is an optional phase for assessment of myocardial infarction (Fig. 5). As with late gadolinium enhancement MRI (LGE-MRI), iodinated contrast is retained in the extracellular space of scar/fibrosis [48]. Like LGE-MRI, LIE-CT has the potential for accurate estimation of myocardial viability [48, 49]. LIE-CT is feasible for the assessment of non-ischemic cardiomyopathy such as cardiac sarcoidosis [50]. A large amount of iodine contrast medium is required to clearly visualize LIE. In the protocol including both CTA and CTP, the amount of contrast is enough for LIE-CT [51, 52], while in the protocol including CTA alone, additional injection of contrast medium might be required for LIE-CT [49, 53]. Previously limited by a low contrast-to-noise ratio, LIE-CT has been improved by recent technological advances, such as use of a low tube voltage and iterative reconstruction with a low radiation dose (≤ 2 mSv) [52]. With DECT, low-energy VMI and the iodine map also improve the visibility of myocardial infarction on LIE-CT [53].

### Interpretation of CTP images

**Fig. 6:**
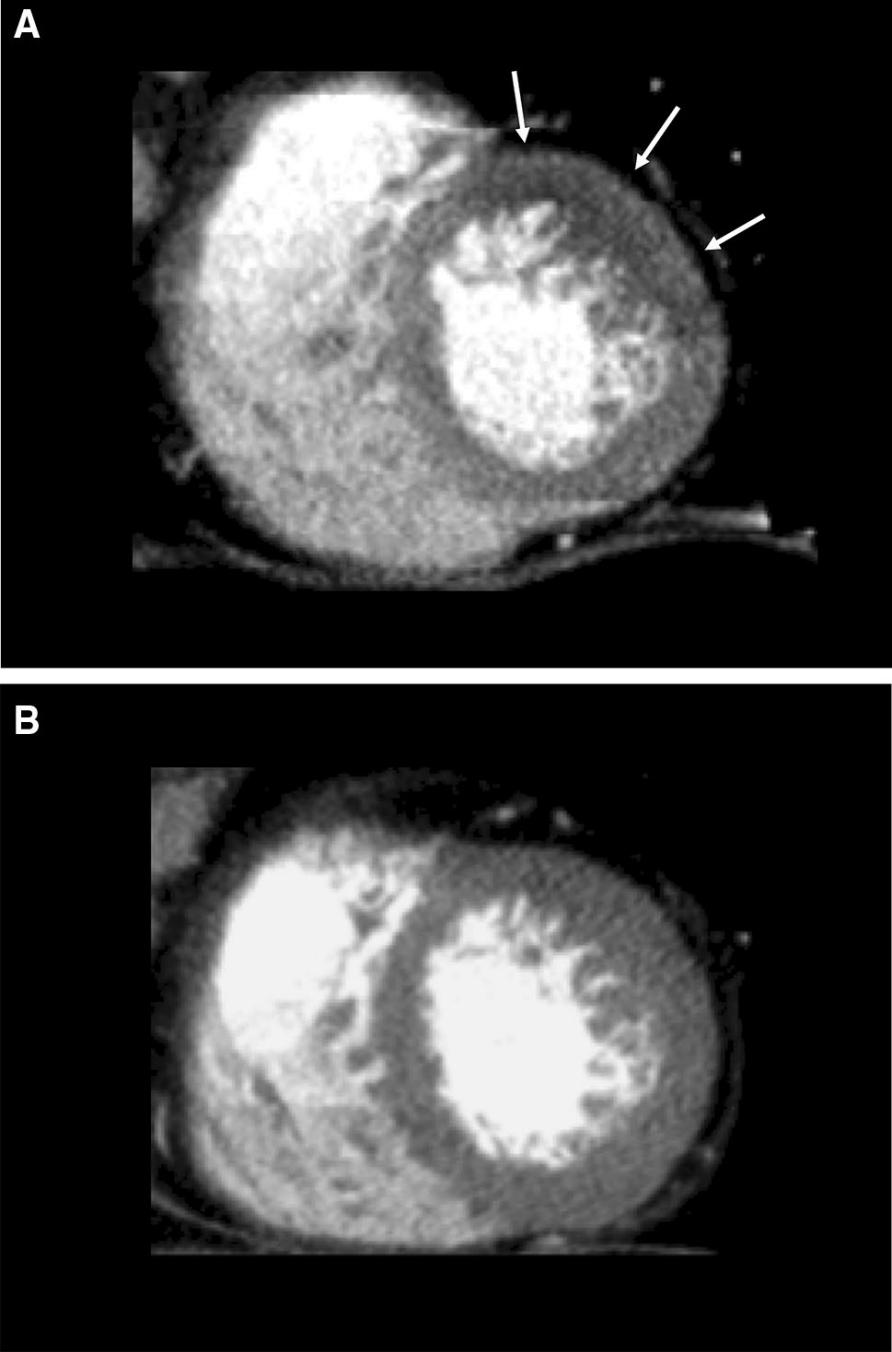
Static CTP scans for single-vessel disease in a 68-year-old woman with chest pain. She had hypertension and dyslipidemia. **a** A short-axis CTP image following stress shows a subendocardial perfusion defect in the mid anterolateral wall (arrows). **b** A rest CTP image in the same position as **a** shows no perfusion defect, consistent with myocardial ischemia. *CTP* computed tomography perfusion

Visual assessment is the main evaluation method for static CTP. A perfusion defect is seen as an area with attenuation that is lower than that of remote myocardium in a subendocardial or transmural distribution. The perfusion defect in ischemia could be seen only on the stress CTP images. The perfusion defect in infarction could be seen on both stress and rest images, but the infarction is sometimes missed in the rest images, especially in stress-first protocol. Therefore, LIE-CT is required to identify the infarction [21]. CTP has higher spatial resolution and diagnostic performance for detecting a perfusion abnormality than single-photon emission tomography (SPECT) [54] (Fig. 6, movie in Online Resource 1). The ischemia can be scored semi-quantitatively at a segmental level, similar to SPECT [55]. The American Heart Association recommends that the left ventricle be divided into 17 segments for regional analysis of myocardial perfusion [56]. The scores of the 17 segments can be added to give the summed stress and rest scores, with their difference being the summed difference score [56].

False positives are seen on CTP due to several types of artifact. The most common is beam-hardening artifact, which is caused by preferential attenuation of low-energy photons in a polychromatic X-ray beam. Beam hardening is usually transmural and always occurs in the plane of the X-ray beam adjacent to highly attenuating structures. On CTP, beam-hardening artifact is caused by dense contrast in the left ventricular cavity and descending aorta adjacent to the free wall of the left ventricle and is most commonly seen in the basal inferior and anterior walls of the left ventricle [21, 55]. These artifacts can be minimized by using dedicated beam-hardening correction algorithms or DECT [47, 57]. False-positive perfusion defects may also be seen due to cardiac or respiratory motion or reconstruction artifacts, such as cone beam artifact. Misalignment artifacts can produce image stacks acquired during different heartbeats [21]. Semi-quantitative perfusion measurements have been developed for static CTP and include the transmural perfusion ratio (TPR) and myocardial perfusion reserve index (MPRI) [58]. The TPR and MPRI are calculated as follows: TPR = subendocardial mean attenuation density (AD)/subepicardial mean AD, where MPRI = (AD stress − AD rest)/AD rest. This semi-quantitative assessment has diagnostic performance comparable to that of visual assessment for detection of obstructive CAD [59].

**Fig. 7:**
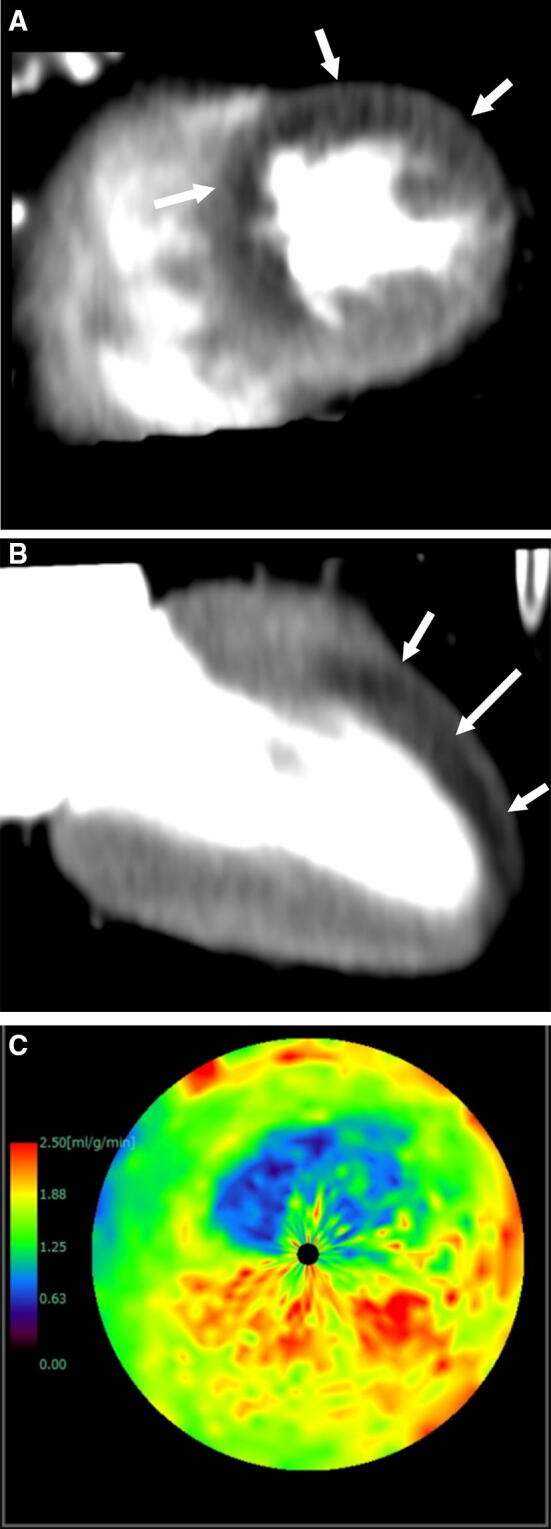
Dynamic CTP scans for single-vessel disease in an 87-year-old man with chest pain. He had hypertension and a history of smoking. Short-axis (**a**) and two-chamber (**b**) grayscale stress dynamic CTP images showing a subendocardial perfusion defect in the anterolateral, anterior, and anteroseptal segments (arrows). **c** A CT-MBF color-coded image shows that the MBF in the ischemic myocardium is lower than in the remote myocardium. *CTP* computed tomography perfusion, *CT-MBF* computed tomography derived-myocardial blood flow

Dynamic CTP can be assessed visually in the same way as static CTP (movie in Online Resource 2). However, dynamic CTP is mainly assessed by a semi-quantitative or fully quantitative technique, because the visual interpretation of CTP image is more difficult than that of MRI due to the lower image contrast [60]. Semi-quantitative parameters are derived from the myocardial time attenuation curve and include upslope, peak enhancement, time to peak, and area under the curve. Upslope has been shown to have the highest diagnostic performance for detection of a perfusion abnormality [61]. Fully quantitative parameters are derived from both arterial and myocardial time attenuation curves, including myocardial blood flow (MBF), myocardial blood volume, and mean transit time. MBF is the most important quantitative parameter and is frequently used for assessment of perfusion whereas the usefulness of myocardial blood volume and mean transit time is not well studied [62] (Fig. 7). Quantitative parameters are useful for assessment of balanced ischemia in triple-vessel disease, which is difficult to assess on SPECT. There are several mathematical methods for quantifying myocardial perfusion in dynamic CTP, including maximum upslope, a compartment model, an extended Toft model, a Patlak plot, a Fermi parametric model, and model-independent deconvolution [35, 63, 64]. However, the optimal quantification method remains controversial and the cut-off value on dynamic CTP imaging is still not standardized. Recently, relative flow reserve has been shown to be a better alternative to absolute MBF [40, 65, 66]. CFR is calculated as a quantitative ratio of stress MBF to rest MBF in stress and rest dynamic CTP protocol [36]. CFR provides the information of not only epi-coronary artery but also microvascular function, and is significantly impaired due to obstructive epicardial CAD or coronary microvascular dysfunction [67]. Obara et al. reported that CFR and stress MBF had sufficient sensitivity, PPV, and NPV to detect obstructive CAD assessed by invasive coronary angiography both in per-patient and in per-vessel analyses as with ^15^O-labelled water PET [68, 69]. Nakamori et al. reported that CFR had the additive value in addition to the stress-rest perfusion MRI for detecting reduced FFR in multivessel disease [70]. van de Hoef et al. reported that discordance between CFR and FFR originated from the involvement of the microvascular function, and the risk for major adverse cardiac events associated with FFR/CFR discordance was mainly attributable to stenoses with abnormal CFR [71]. Although CFR is very useful as diagnostic and prognostic predictor, stress and rest dynamic CTP protocol has restriction of use due to the radiation exposure. Therefore, further radiation reduction of dynamic CTP is required to use CFR in clinical routine.

### Iterative reconstruction and other algorithms for CTP

Radiation exposure and suboptimal image contrast are important concerns in CTP imaging. Iterative reconstruction algorithms decrease image noise, allowing use of low-dose CT techniques (e.g., low tube voltage) with maintenance of image quality [72, 73]. There are several iterative reconstruction (IR) algorithms; some are hybrid IR” techniques that are combined with filtered back projection (FBP) to reduce reconstruction time and some are “fully IR” with higher performance [74]. Use of a fully IR technique improves image quality without altering hemodynamic parameters on low-dose dynamic CTP when compared with FBP or hybrid IR [39]. Recently, artificial intelligence was used to improve CT image reconstruction [75]. These techniques have the potential to optimize the quality of low-dose myocardial CTP images with shortening of reconstruction times [76, 77].

### Advantages of CTP

**Fig. 8:**
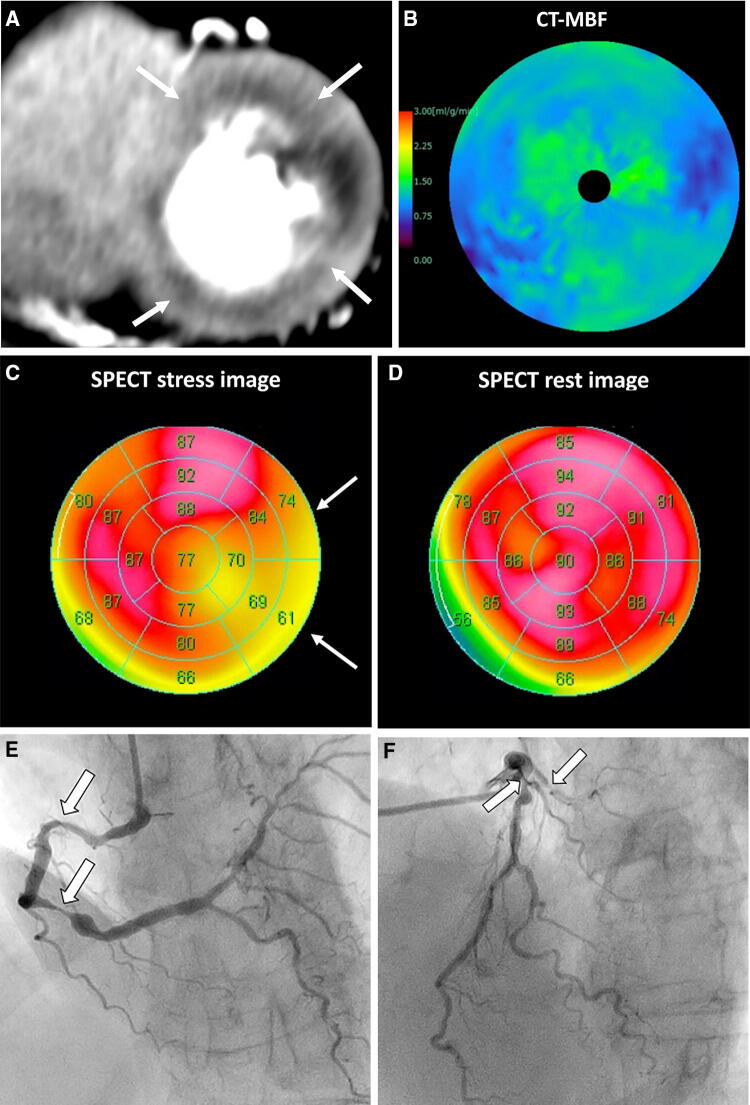
Dynamic CTP scans for triple-vessel disease in an 84-year-old woman with chest pain. **a** Short-axis view of a dynamic CTP (grayscale) shows a subendocardial perfusion defect in the entire circumference of the heart (white arrows). **b** A CT-MBF color-coded image shows low CT-MBF throughout the heart. SPECT during **c** stress and **d** at rest shows a reversible perfusion defect in the lateral wall but no marked perfusion defect in the other regions. Invasive coronary angiography of the **e** right and **f** left coronary arteries shows severe triple-vessel disease. In this case, single-photon emission tomography did not accurately detect the presence of triple-vessel disease, known as balanced ischemia. *CTP* computed tomography perfusion, *CT-MBF* computed tomography derived-myocardial blood flow, *SPECT* single-photon emission computed tomography

A major advantage of CTP is that it allows a perfusion defect to be visualized directly with high temporal and spatial resolution, as would MRI [78]. CTP is useful for assessment of the hemodynamic significance and effective classification of coronary lesions, allowing integrated evaluation of CAD when combined with CTA (Fig. 8) [30, 79]. Moreover, myocardial perfusion can be quantified and the effect of treatment after revascularization can be evaluated [35, 80]. CTP can provide functional information even in patients with heavy calcifications or stenting, which are limitations of CT-FFR [41, 81]. CTP is preferred by patients [82] and is more cost-effective than SPECT [83].

### Limitations of CTP

CTP is not widely available because it requires a high level of expertise and multiple resources, including advanced scanners and reconstruction algorithms. Greater radiation exposure, use of a higher contrast dose compared with CTA alone (static CTP alone 1.9–15.7 mSv, dynamic CTP alone 3.8–12.8 mSv, combined CTA and stress CTP protocol 3–16 mSv) [37, 84], and side effects of medications are also issues. Static CTP is limited in evaluation of patients after CABG because of the complexity of myocardial perfusion via the native coronary arteries and bypass grafts.

### Current evidence of CTP

Multiple studies have established the high diagnostic performance of both static and dynamic CTP for detecting hemodynamically significant stenosis [34, 36, 41, 61, 62, 78, 81, 84–90] (Table 2). Recent meta-analyses indicated that dynamic CTP has higher sensitivity but lower specificity than static CTP (sensitivity, 85% vs 72–80%; specificity, 90–93% vs 81–83%) [84, 90] (Table 2). Moreover, the CORE320 study (*n* = 381) demonstrated that static CTP has incremental diagnostic value over CTA for detection of hemodynamically significant coronary lesions, with a specificity of 74% vs 51% for CTA, a PPV of 65% vs 53%, and an area under the receiver-operating characteristic curve (AUC) of 0.87 vs 0.84 [91]. Pontone et al. (*n* = 147) reported similar results in static CTP (a specificity of 95% vs 76% for CTA and a PPV of 87% vs 61%) [92]. The CRESCENT-II trial indicated that incorporation of dynamic CTP imaging as part of a tiered diagnostic approach could improve the clinical value and efficiency of cardiac CT in the diagnostic work-up of patients with stable CAD and was an effective alternative to standard guideline-directed functional testing [93]. Both static and dynamic CTP has also been reported to have incremental predictive value over clinical risk factors and CTA in assessment for future major adverse cardiac events (MACE) [94, 95]. Dynamic CTP was shown to have higher prognostic value for MACE than CTA and CT-FFR, independent of clinical risk factors [96].

**Table 2:** Diagnostic performance of computed tomography perfusion with invasive fractional flow reserve as the gold standard

Study	Patients, *n*	Technique	Sensitivity (%)	Specificity (%)	PPV (%)	NPV (%)	AUC	MBF cut-off
Bettencourt et al. [78]	101	Static	55	95	78	87	0.75	
Yang et al. [86]	75	Static	80	95	92	87	0.87	
Yang et al. [87]	72	Static	79	91	86	87	0.88	
Ihdayhid et al. [41]	46	Static	54	92	79	77	0.72	
Greif et al. [62]	65	Dynamic	95	74	49	98	0.71	0.75 ml/g/min
Huber et al. [61]	32	Dynamic	76	100	100	91	0.86	1.64 ml/g/min
Rossi et al. [88]	80	Dynamic	88	90	77	95	0.95	0.78 ml/g/min
Coenen et al. [34]	43	Dynamic	75	78	78	75	0.78	0.76 ml/g/min
Coenen et al. [81]	74	Dynamic	75	61	63	73	0.78	0.91 ml/g/min

Meta-analysis	Vessels, *n*	Protocol	Sensitivity (%)	Specificity (%)	PLR	NLR	AUC
Takx et al. [85]	1074	Static	78	86	5.74	0.22	0.91
Lu et al. [89]	697	Dynamic	85	81	4.46	0.21	0.91
Celeng et al. [90]	2118	Overall	81	86	6.28	0.23	
		Static	72	90			
		Dynamic	85	81			
Hamon et al. [84]	2336	Overall	82	89	7.72	0.21	0.94
		Static	80	93	10.77	0.23	0.96
		Dynamic	85	83	4.89	0.17	0.94

*AUC* area under the curve, *MBF* myocardial blood flow, *NLR* negative likelihood ratio, *NPV* negative predictive value, *PLR* positive likelihood ratio, *PPV* positive predictive value

## CT-fractional flow reserve

### Principle of CT-FFR

CT-FFR calculates the FFR from coronary CTA data at rest using computational fluid dynamics to generate a mathematical model of coronary flow, pressure, and resistance [97]. This modeling relies on the following four important principles and assumptions: (1) under resting conditions, the total coronary flow is proportional to myocardial mass; (2) resting coronary microvascular resistance is inversely proportional to the size of the epicardial coronary arteries; (3) the dilatory response of the coronary arteries to adenosine during ICA is predictable and can be used to create a computational model of the maximal hyperemic state, which is generally simulated by reducing the microvascular resistance by a factor of 0.21, and although adenosine is not required, administration of nitroglycerine is a pre-requisite for measurement of CT-FFR; and (4) solving the complex three-dimensional Navier–Stokes equation that governs fluid dynamics can compute the flow and pressure across the coronary vascular bed [98].

### Technology of CT-FFR

**Fig. 9:**
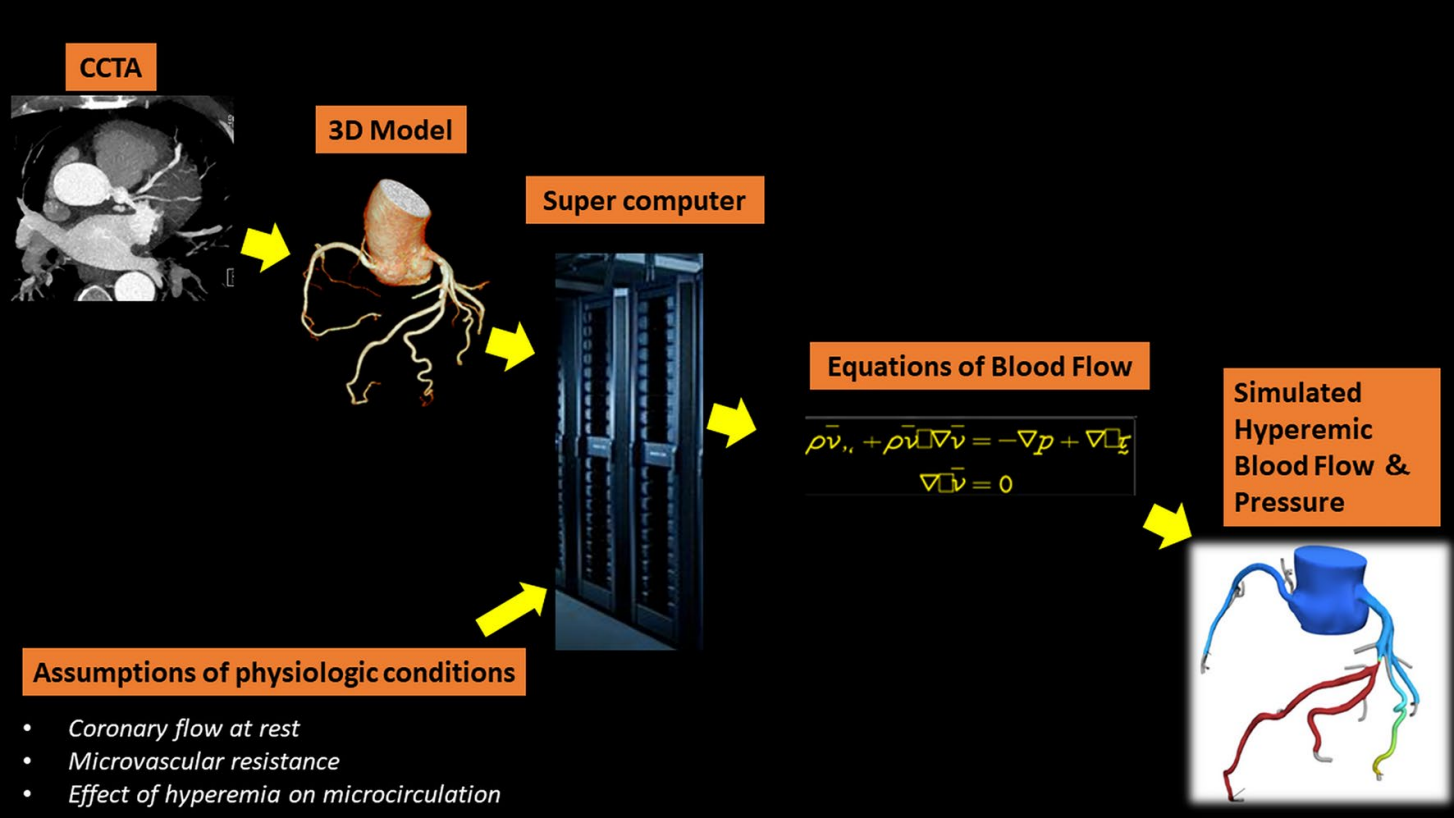
Illustration showing the CT-FFR technique. The CCTA data are segmented and a three-dimensional model is generated. This model is then processed by a supercomputer using assumptions of physiological conditions to solve the Navier–Stokes equation and generate a hyperemic model of coronary flow and pressure. *CCTA* coronary computed tomography angiography, *CT* computed tomography, *FFR* fractional flow reserve

Several large-scale multicenter trials have shown the high diagnostic performance of CT-FFR by a remote analysis service in selected patients with CAD [98–100]. In this technology, the CTA data are transferred through a secure network to an off-site location. A patient-specific three-dimensional model of the coronary arteries is created after segmentation of the CTA data. Using the above-mentioned mathematical assumptions, a supercomputer performs complex post-processing to solve the equations governing fluid dynamics and blood flow [97, 101]. Simulated hyperemic blood flow and pressure data are then generated, and the results are sent back to the referring institution within a few hours (Fig. 9). On-site vendor-based platforms are also available in some institutions, including a machine learning-based algorithm [102, 103], four-dimensional CT image tracking (registration) and structural and fluid analysis [104, 105], and patient-specific lumped parameter models [106, 107].

However, the off-site CT-FFR using a remote analysis service is recently received with national reimbursement approval in Japan, but the available facilities are strictly limited by requirements. Meanwhile, the on-site CT-FFR is also available only for clinical research.

### Interpretation of CT-FFR

**Fig. 10:**
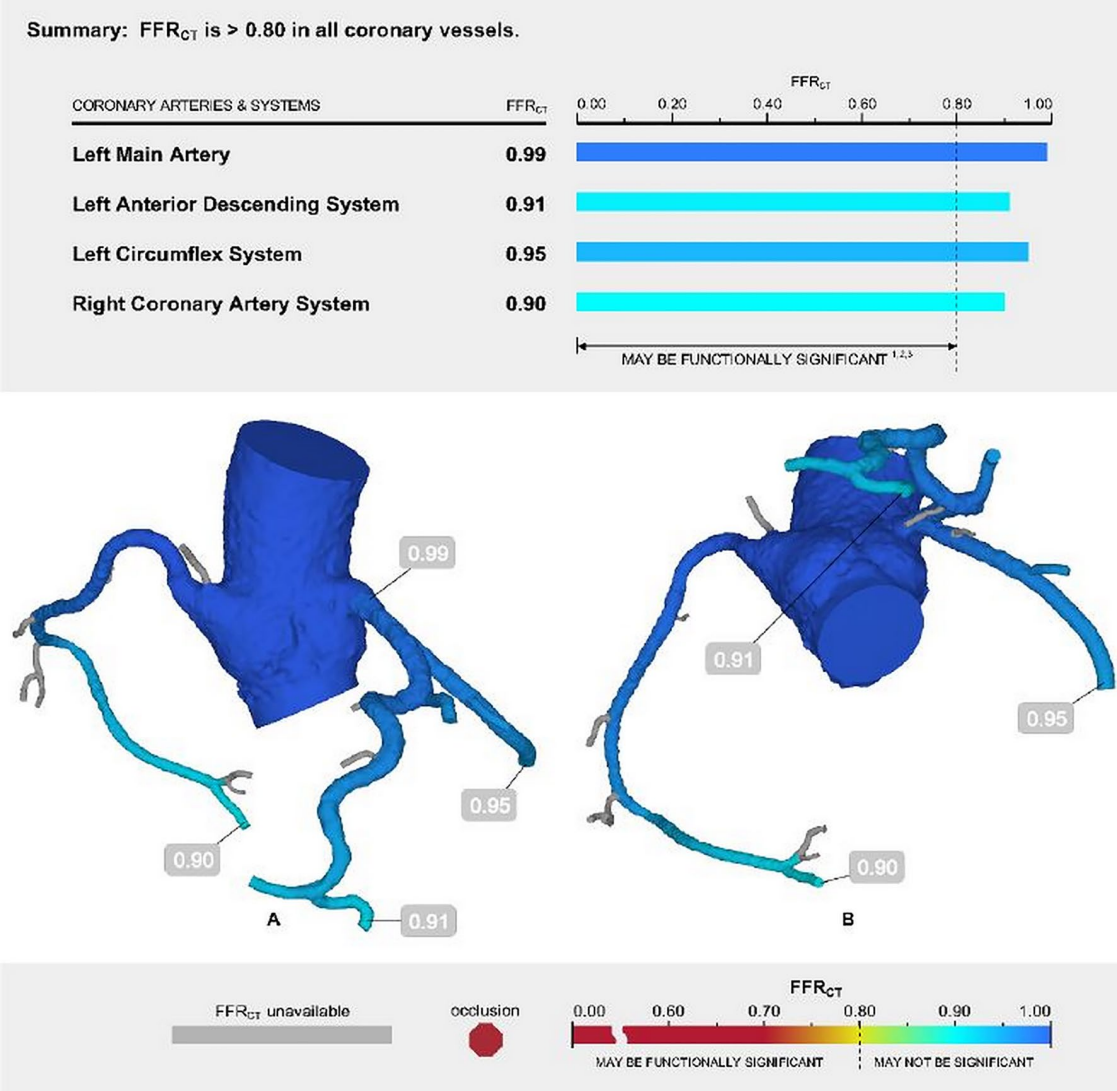
Normal CT-FFR report for a 59-year-old man with atypical chest pain. The CT-FFR report shows the coronary arteries color-coded according to their CT-FFR values. In this patient, all the major coronary arteries show normal values (> 0.8). *CT* computed tomography, *FFR* fractional flow reserve

CT-FFR is presented as a color-coded map of continuous CT-FFR values computed along each coronary vessel (Fig. 10). These values are both specific for a lesion and for the entire coronary tree. CT-FFR results are interpreted in conjunction with anatomic CTA findings, including vessel size, presence and location of stenosis, suitability for revascularization, and other CT-FFR values. Coronary stenosis with a precipitous drop in CT-FFR across the lesion, particularly if < 0.75, is associated with lesion-specific ischemia. A CT-FFR value > 0.8 distal to a stenosis is rarely associated with ischemia. A CT-FFR value between 0.75 and 0.80 is “gray zone” or borderline [101]. It is important to measure the CT-FFR immediately distal to a stenotic vessel because the CT-FFR value in the most distal vessel segment may not necessarily correlate with the functional significance of the stenosis. A gradual drop in pressure along the length of the vessel without focal stenosis, particularly for borderline values, can be a normal phenomenon or due to a small vessel size, inadequate response to a nitrate, diffuse disease, or a serial lesion [97, 101]. There are ICA data showing that diffuse disease can cause a hemodynamically significant drop in the pressure gradient [108] but the CT data are inadequate. The referring clinician should interpret the findings in view of the anatomy, entire physiological model, and symptoms.

### Clinical utility: when to use CT-FFR?

**Fig. 11:**
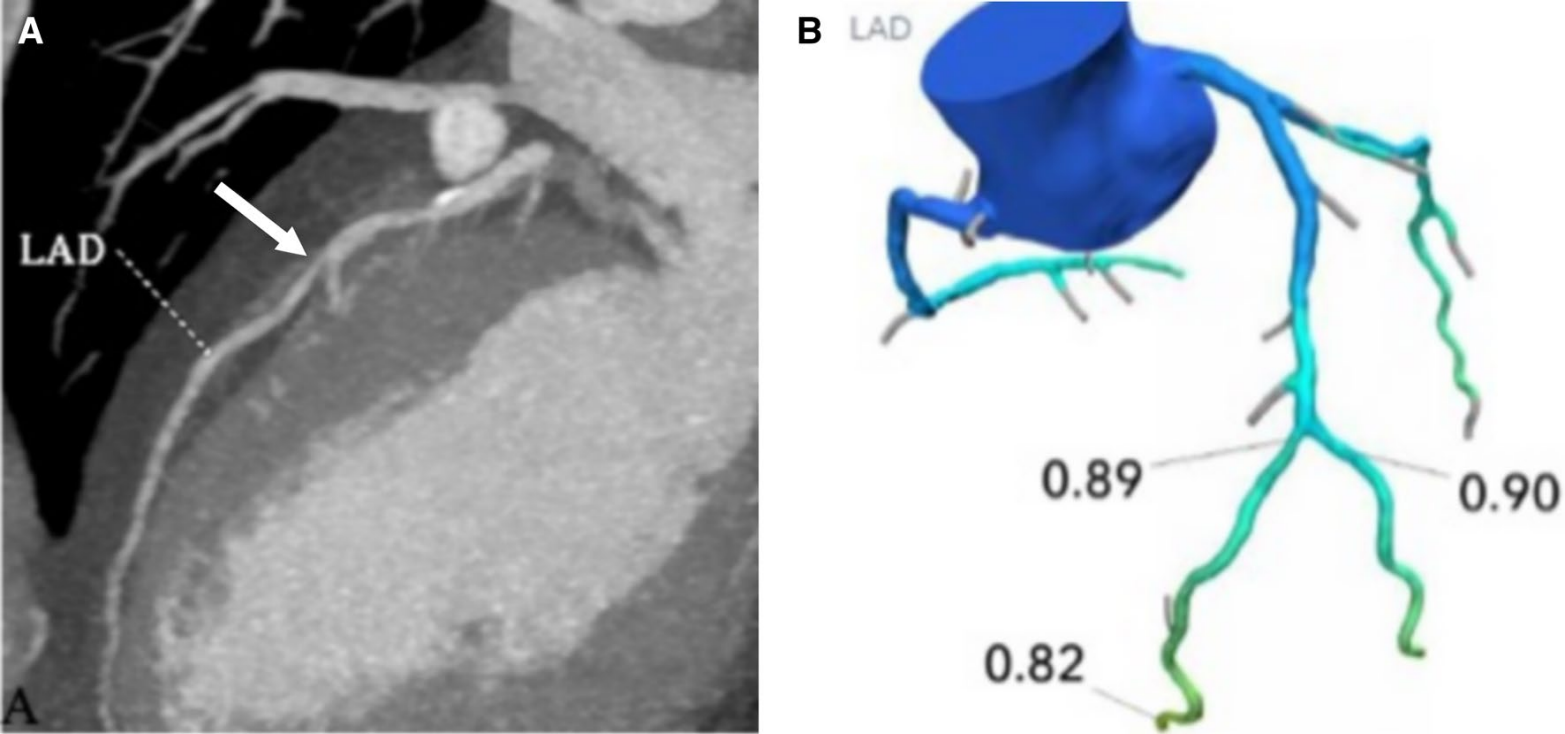
**a** A curved multiplanar reconstruction image of the LAD in a 52-year-old man with chest pain showing moderate stenosis of the mid LAD (arrow). **b** CT-FFR in the same patient shows a value of 0.89 in the mid LAD, which is within normal limits, indicating that there is no lesion-specific ischemia. The patient was referred for medical management. Therefore, CT-FFR helped to avoid ICA. *CT* computed tomography, *FFR* fractional flow reserve, *ICA* invasive coronary angiography, *LAD* left anterior descending artery

**Fig. 12:**
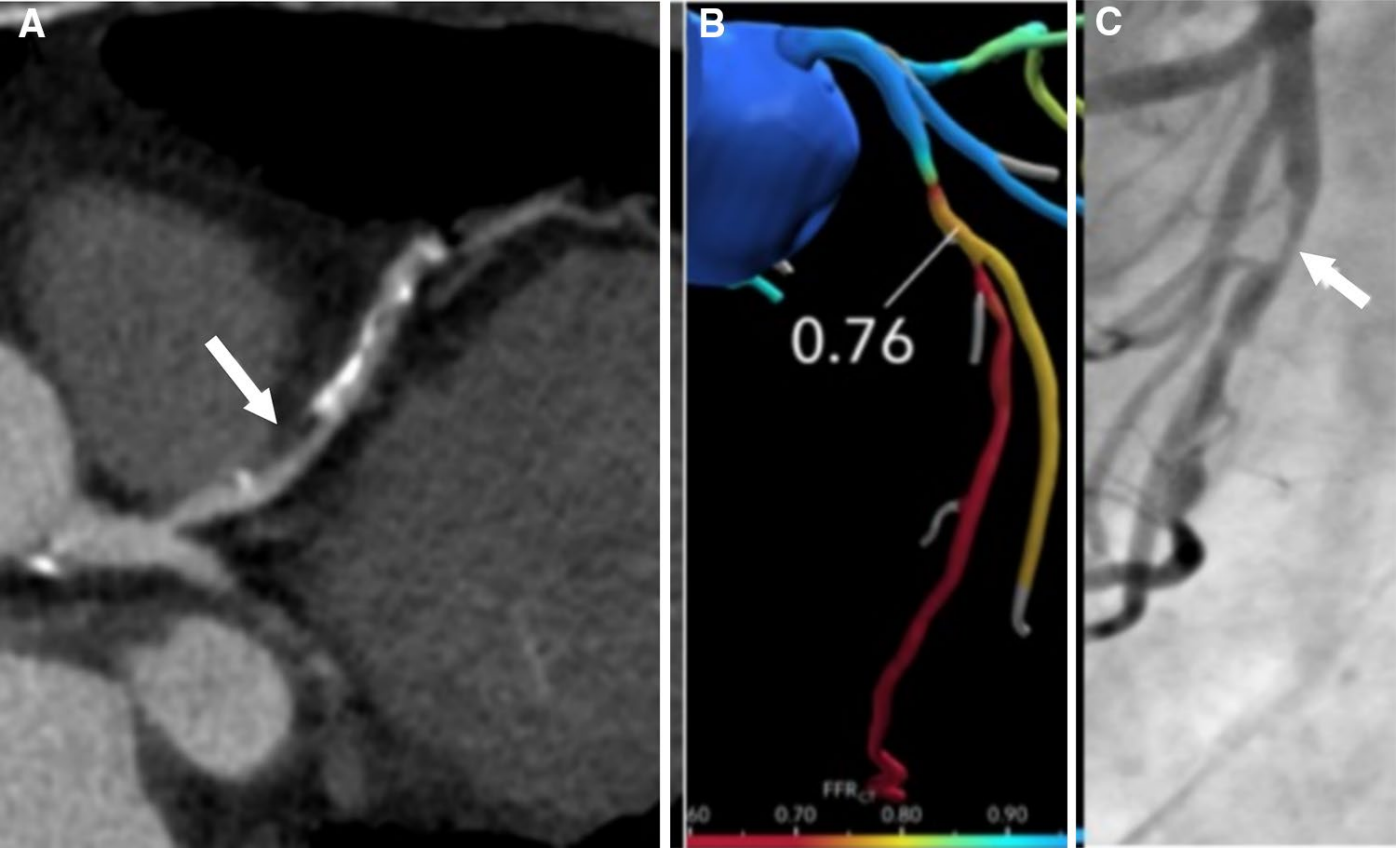
**a** A curved multiplanar reconstruction image of the LAD in a 66-year-old woman showing a non-calcified plaque in the proximal LAD causing moderate luminal stenosis (arrow). **b** CT-FFR shows a value of 0.76 in the proximal LAD, which is indicative of hemodynamically significant stenosis. **c** The patient was referred for ICA, which confirmed a significant stenosis with a decreased FFR of 0.65. *CT* computed tomography, *FFR* fractional flow reserve, *ICA* invasive coronary angiography, *LAD* left anterior descending artery

CT-FFR is most valuable in patients with moderate (50–70%) stenosis on CTA. CT-FFR clarifies the hemodynamic significance of stenosis in these patients and aids decision-making [109]. If a patient has moderate coronary artery stenosis on CTA and the CT-FFR value is > 0.8, ICA can be avoided (Fig. 11). However, if the value is < 0.8, ICA can be performed with the intention to treat the lesion (Fig. 12). If CTA shows a non-obstructive lesion (< 50% stenosis), the patient is referred for medical treatment and there is no need to perform CT-FFR or ICA. If CTA shows severe stenosis, the patient is referred for ICA without the need for CT-FFR. In patients with multivessel disease or tandem lesions, CT-FFR helps to identify the lesions needing revascularization [110]. However, CT-FFR may underestimate the contribution of true stenosis in serial stenoses [111]. A noninvasive PCI planning tool has been devised to help determine the contribution of true stenosis in serial and diffuse CAD [111]. A virtual stent can also be placed and the response to revascularization estimated [112].

### Current evidence of CT-FFR

The accuracy of CT-FFR has been validated in several studies (Table 3) [81, 98–100, 103, 105–107, 113–122], predominantly with off-site technology, which uses invasive FFR as the gold standard. The specificity of CT when CT-FFR is used is better than when CTA alone is used [82% vs 40% in DISCOVER-FLOW (per-vessel), 54% vs 42% in DEFACTO (per-patient), and 79% vs 34% in NXT (per-patient)] [98–100]. Similar results were seen with on-site vendor-based and machine learning algorithms [81, 103, 105–107, 113, 116–119]. One meta-analysis showed a pooled specificity of 76% and an odds ratio of 26.2 for detecting ischemic lesions on the per-patient basis [121], whereas another study showed that CT-FFR had higher specificity than CTA (71% vs 39%) but similar sensitivity (91%) on the per-patient basis [122]. CT-FFR had diagnostic accuracy similar to that of SPECT but had higher sensitivity for predicting FFR-guided revascularization [114]. With good-quality CTA images, CT-FFR was found to have higher diagnostic performance than CTA, SPECT, or PET on a per-vessel basis whereas PET had favorable performance on per-patient and intention-to-diagnose analysis [115].

**Table 3:** Diagnostic performance of computed tomography-fractional flow reserve with invasive fractional flow reserve as the gold standard

Study	System	Study type	Basis	Accuracy (%)	Sensitivity (%)	Specificity (%)	PPV (%)	NPV (%)	AUC
Koo et al. (DISCOVER-FLOW) [98]	Off-site	Prospective multicenter	159 vessels	84	88	82	74	92	0.90
Min et al. [DEFACTO) [99]	Off-site	Prospective multicenter	407 vessels	69	80	63	56	84	NA
Nørgaard et al. (NXT) [100]	Off-site	Prospective multicenter	484 vessels	86	84	86	61	95	0.93
Sand et al. (ReASSESS) [114]	Off-site	Prospective single-center	143 patients	70	91	55	58	90	NA
Driessen et al. [115]	Off-site	Prospective single-center	505 vessels	87	90	86	65	96	0.94
Coenen et al. [81]	On-site	Retrospective two-center	142 vessels	70	82	60	65	79	0.78
De Geer et al. [116]	On-site	Retrospective single-center	23 vessels	78	83	76	56	93	NA
Fujimoto et al. [105]	On-site	Retrospective two-center	104 vessels	84	91	78	76	92	0.85
Donnelly et al. [106]	On-site	Prospective two-center	60 vessels	78	91	72	63	93	0.89
Kim et al. [117]	Off-site	Prospective multicenter	48 vessels	77	85	57	83	62	NA
Renker et al. [113]	On-site	Retrospective single center	67 vessels	85	85	85	71	93	0.92
Wardziak et al. [118]	On-site	Retrospective single center	96 vessels	74	76	72	67	80	0.84
van Hamersvelt et al. [107]	On-site	Retrospective single-center	77 vessels	83	89	78	79	89	0.87
Coenen et al. [103]	On-site	Retrospective multicenter	525 vessels	78	81	76	70	85	0.84
Kurata A et al. [119]	On-site	Retrospective multicenter	91 vessels	82	89	75	79	87	0.91

Meta-analysis	Total number of studies	Vessels	DOR	Sensitivity (%)	Specificity (%)	PLR	NLR	AUC
Baumann et al. [120]	5	1306	NA	84	75	NA	NA	0.90
Wu et al. [121]	7	1377	16.87	84	76	3.51	0.21	0.86
Danad et al. [122]	3	1050	19.15	83	78	4.02	0.22	0.92

*AUC* area under the curve, *DOR* diagnostic odds ratio, *FFR* fractional flow reserve, *NA* not available, *NLR* negative likelihood ratio, *NPV* negative predictive value, *PLR* positive likelihood ratio, *PPV* positive predictive value

The studies for CT-FFR are summarized in Table 4. The ability of CT-FFR to evaluate lesion-specific ischemia with high specificity makes it an effective gatekeeper for ICA. In NXT, 68% of false positives were reclassified as true negatives [100]. PLATFORM, which was a clinical utility trial, showed that use of CT-FFR resulted in cancellation of ICA in 61% of patients in whom it had been planned based only on CTA findings without any adverse events at the 90-day follow-up [123]. By using CT-FFR, the overall incidence of non-obstructive disease on ICA decreased to 12% from 73% (a decrease of 61%) [123]. At the 12-month follow-up, adverse events were infrequent (only 5 of 581 followed cases). In the planned invasive stratum, mean costs were 33% lower with CTA and CT-FFR [124]. Data are also emerging on the use of CT-FFR in decision-making and outcomes. The FFR_CT_ RIPCORD study showed that use of CT-FFR resulted in a change in the management plan (medical vs PCI vs CABG) in 36% of patients. There was a 30% reduction in PCI, an 18% change in the target vessel, and reassignment from optimal medical therapy to PCI in 12% of cases [125]. The SYNTAX III Revolution trial showed that CT-FFR aids decision-making without need for ICA in patients with left main or 3-vessel CAD. A decision-making based on CTA and CT-FFR showed high agreement with the decision derived from ICA [126]. This study also showed that the addition of CT-FFR to CTA alone changed the treatment decision (between PCI and CABG) in 7% of the patients and modified selection of vessels for revascularization in 12% [127]. Using CT-FFR resulted in 12% fewer MACE at 1 year and 30% lower costs with improved quality of life in comparison with ICA and visual guidance [128]. There was no MACE at 1 year in patients in whom ICA was deferred as a consequence of a negative CT-FFR, indicating that this is a safe and feasible test [113, 125]. The 1-year outcomes in the ADVANCE FFR_CT_ registry study also indicated a low event rate, fewer MACE, and less revascularization in those with negative CT-FFR [129].

**Table 4:** Summery of the studies and the trials for computed tomography-fractional flow reserve

Study/trial	Objectives	Study type	Results
DISCOVER-FLOW study [98]	Diagnostic performance (CT-FFR vs CTA)	Prospective Multicenter	A higher AUC of CT-FFR than CTA (per-patient and per-vessel) Good correlation of CT-FFR values with FFR values
DeFACTO study [99]	Diagnostic performance (CT-FFR vs CTA)	Prospective Multicenter	A higher AUC of CT-FFR than CTA (per-patient)
NXT trial [100]	Diagnostic performance (CT-FFR vs CTA)	Prospective Multicenter	A higher accuracy of CT-FFR than CTA (per-patient and per-vessel) A higher AUC of CT-FFR than CTA (per-patient and per-vessel)
PLATFORM trial [123, 124]	The clinical, economic, and quality-of-life outcomes of using CT-FFR instead of usual care	Prospective Multicenter	61% cancellation of ICA with no adverse events at the 90-day follow-up Infrequency of adverse events at the 12-month follow-up 33% lower costs with CT-FFR in the ICA planned patients
FFR_CT_ RIPCORD study [125]	The effect of adding CT-FFR to CTA alone for assessment of severity and patient management in patients with stable chest pain	Retrospective Multicenter (dataset from NXT study)	30% reduction in PCI, an 18% change in the target vessel Reassignment from OMT to PCI in 12% of cases
SYNTAX III Revolution trial [126, 127]	The feasibility of decision-making and treatment planning based only on non-invasive imaging in patients with LMT or 3VD	Prospective Multicenter	Decision-making with CTA and CT-FFR was feasible 7% change in the treatment recommendation, 12% change in the target vessels (addition of CT-FFR to CTA alone)
ADVANCE FFR_CT_ study [129]	Real-world clinical utility on decision-making of CT-FFR in patients with symptoms concerning for CAD	Prospective Multicenter	Low event rate, fewer MACE, and less revascularization in patients with negative CT-FFR at the 12-month follow-up
PERFECTION study [91]	Diagnostic performance (CTA + CT-FFR vs CTA + static CTP)	Prospective Multicenter	Better diagnostic performance of both CTA + CT-FFR and CTA + static CTP than CTA alone (Specificity, PPV, and AUC) No differences between CTA + CT-FFR vs CTA + static CTP

*AUC* area under the curve, *CAD* coronary artery disease, *CTA* computed tomography angiography, *CT-FFR* computed tomography–derived fractional flow reserve, *CTP* computed tomography perfusion, *ICA* invasive coronary angiography, *LMT* left main trunk, *MACE* major adverse cardiac events, *OMT* optimal medical therapy, *PPV* positive predictive value, *PCI* percutaneous coronary intervention, *3VD* triple-vessel disease

CT-FFR shows promise in the evaluation of biomechanical forces on atherosclerotic plaques, which play a role in their development and progression [130, 131]. CT-FFR can also aid in the prediction of acute coronary syndrome with potentially superior accuracy (area under the curve, 0.725) on CTA [132].

### Limitations of CT-FFR

CT-FFR relies on high-quality CTA images. Therefore, it is critical to adhere to a rigorous guideline-driven protocol [133] with maximal coronary vasodilation and without image noise, motion, or misalignment artifacts [101, 134]. Inadequate contrast opacification and calcium blooming can also negatively affect CT-FFR evaluation [135]. However, recent studies have shown that the performance of CT-FFR in patients with high coronary calcium score is superior to that of CTA alone [130, 136]. The rates of rejection on CT-FFR analysis in the ADVANCE registry and in a large clinical cohort were 2.9% and 8.4%, respectively [137]. CT-FFR is currently not suitable for patients with stenting or CABG. CT-FFR is also not effective in patients with unstable angina and performs modestly in detecting ischemia in non-culprit lesions in patients with STEMI, probably because of the vessel volume is smaller in these patients than in those with stable angina, which confounds the assumption that size is related to resistance [138]. As of now, CT-FFR is not ideal for use in the emergency setting because of the above-mentioned inadequate assumptions. A recent study showed that although CT-FFR has good accuracy overall (81.0%), it has poor accuracy (46.1%) in the borderline CT-FFR range (0.7–0.8) [139]. Therefore, the results of CT-FFR should be interpreted cautiously in the context of patient-specific risk factors. Furthermore, there are data that show CT-FFR to be abnormal in up to 16.6% of patients with insignificant stenosis (< 50%) and conversely that it can be normal in 50% of patients with moderate stenosis [140, 141]. Recent studies have cast doubt on the reproducibility of CT-FFR [138]. For example, there is not a perfect match between the CT-FFR and invasive FFR values [139]. This could be because the pressure sensor on invasive FFR is not at the exact location where CT-FFR was measured [101] or the dose of nitroglycerin before CTA was inadequate or nitroglycerin was not administered at all [101].

## Which CT imaging technique is best for ischemia?

**Fig. 13:**
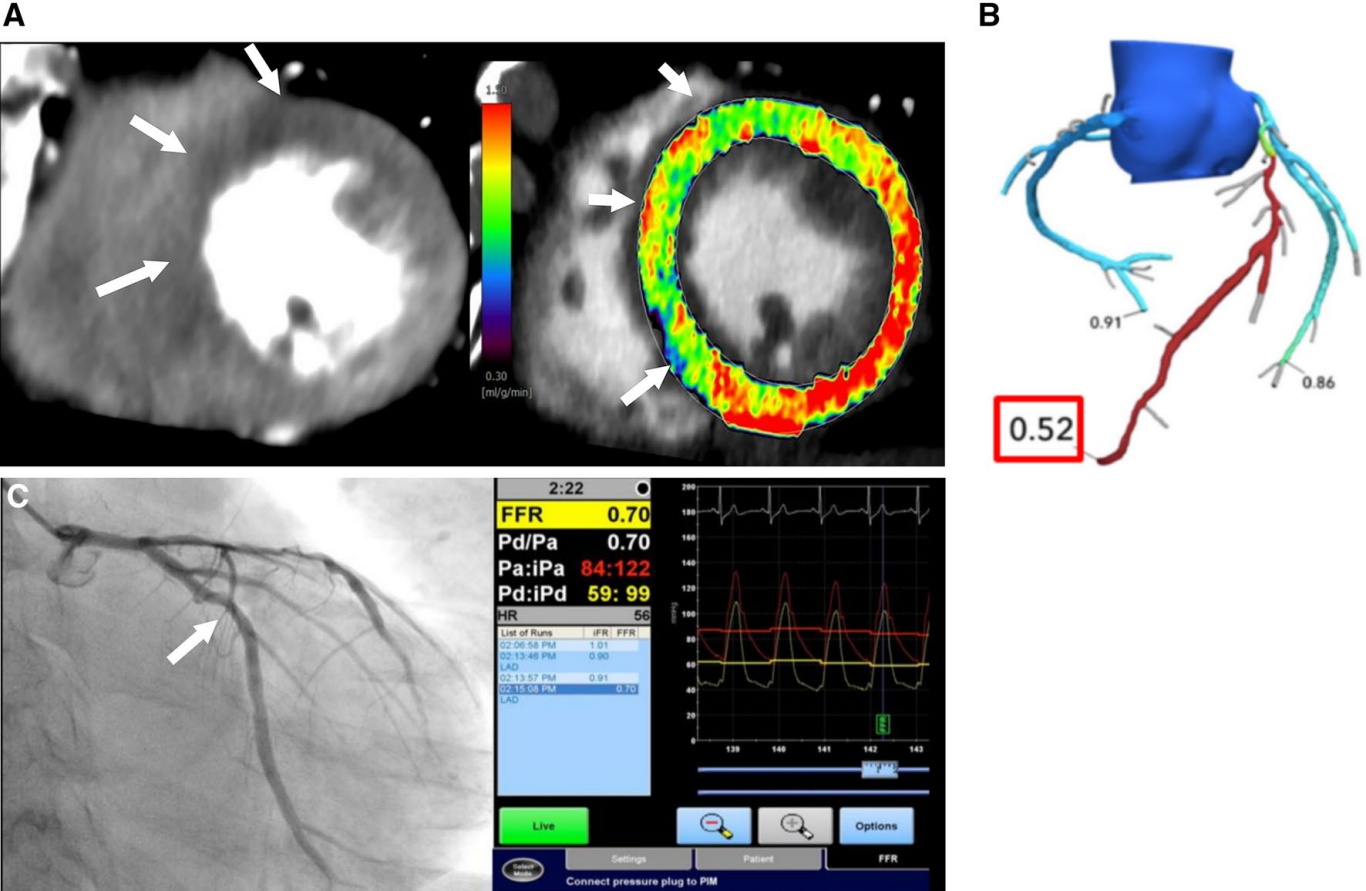
CTP versus CT-FFR. **a** CTP image and CT-MBF map showing a perfusion defect and lower CT-MBF in the anteroseptal wall (arrows). **b** A CT-FFR image in the same patient showing abnormal CT-FFR with a low value of 0.52 at the LAD. **c** ICA showing a stenotic lesion in the mid LAD with an abnormal FFR of 0.70. *CTP* computed tomography perfusion, *CT-MBF* computed tomography derived-myocardial blood flow, *CT* computed tomography, *FFR* fractional flow reserve, *ICA* invasive coronary angiography, *LAD* left anterior descending artery

The choice between CTP and CT-FFR depends on the availability of the technology and expertise (Table 5). CTP requires a high-end scanner, ideally with single heartbeat coverage and dedicated personnel, protocols, and post-processing. CT-FFR can be obtained from CTA data from any scanner, but the images must be of high quality, the results are not available immediately, and there is an additional cost for using off-site CT-FFR technology [142]. On-site systems are not commercially available as yet. CT-FFR is better than CTP for identifying patients with balanced ischemia, multi-vessel disease, or serial lesions who will benefit from revascularization [98] but is of limited value in patients with unstable angina, especially for non-culprit lesions in those with a recent STEMI. CTP is better than CT-FFR in patients with heavy calcifications or stenting [143, 144]. Coenen et al. showed that the diagnostic performance of CTP and CT-FFR was comparable for identifying functionally significant CAD assessed by invasive FFR [81] (Fig. 13). A similar study showed that CT-FFR and CTP had comparable performance, with either test improving the performance of CTA [87]. A meta-analysis also showed that the pooled specificity values for CTP (0.77) and CT-FFR (0.72) were higher than that of CTA (0.43) [145]. Otherwise, Li et al. showed that CTP outperformed CT-FFR for identifying lesions causing ischemia assessed by invasive coronary angiography and FFR [146]. The PERFECTION study showed that both CTA + CT-FFR and CTA + CTP have better diagnostic performance than CTA alone [92]. Integrated CTP and CT-FFR has better performance than either of these techniques used alone [81].

**Table 5:** Comparison of the various computed tomography techniques used to evaluate myocardial ischemia

	Advantages	Disadvantages
Coronary CTA	Visualization of coronary artery stenosis and plaque morphology	Unassessable segments (artifact, calcification)
	Widely available in clinical practice	Low PPV for detecting myocardial ischemia
CT-FFR	CTA anatomy- and CFD-based functional assessment	Depends on image quality of coronary CTA
	No scan additional to coronary CTA	Appropriate patient selection (image-related, patient-related factors influencing CT-FFR calculation)
	High diagnostic performance	Remote service (time-consuming)
	Effective modification to coronary CTA based decision-making	On-site analysis (requiring a learning period, objectivity)
		Less information on the stenosis-related territory
CTP	High spatial resolution	Radiation exposure and contrast dose additional to coronary CTA
	Real-time stress myocardial perfusion imaging	Risk of side effects from the vasodilator agent
	Visualization of myocardial ischemia (area and transmural extent)	Long examination time (30–60 min)
	Quantification (CT-MBF using dynamic CTP)	
	Incremental value to coronary CTA	

*CFD* computational flow dynamics, *CTA* computed tomography angiography, *CT-FFR* computed tomography-derived fractional flow reserve, *CT-MBF* computed tomography derived myocardial blood flow, *CTP* computed tomography perfusion, *PCI* percutaneous coronary intervention

## Conclusion

Coronary CTA can exclude CAD with a high degree of certainty but has limited ability to evaluate the hemodynamic significance of stenosis because of its poor specificity. Emerging technologies such as CT-perfusion and CT-FFR can provide information on the hemodynamic significance of stenosis, which expands the capabilities of CT. These techniques make CT valuable for risk stratification and decision-making in patients with myocardial ischemia.

## Electronic supplementary material

Below is the link to the electronic supplementary material.

Supplementary file1 Online Resource 1 A retrospective ECG-gated static myocardial CTP image in two-chamber, four-chamber, and short-axis planes following stress in a 73-year-old man showing a subendocardial perfusion defect in the apical, mid anterior, and anteroseptal segments (A). There is also hypokinesis in these segments (A). A retrospective ECG-gated static myocardial CTP image at rest in the same planes shows no abnormality in perfusion or wall motion (B). These findings are consistent with myocardial ischemia (B). CTP, computed tomography perfusion (MP4 248 kb)

Supplementary file2 (MP4 312 kb)

Supplementary file3 Online Resource 2 A dynamic myocardial CTP image following stress in the short-axis plane in a 69-year-old woman showing perfusion defects in the mid lateral and inferior walls (A). A dynamic myocardial CTP image at rest in the same patient showing that the perfusion defect in the lateral wall is reversible, which is consistent with ischemia in the left circumflex territory (B). The perfusion defect in the inferior wall is fixed, consistent with an infarction in the right coronary artery territory (B). CTP, computed tomography perfusion (MOV 508 kb)

Supplementary file4 (MOV 526 kb)
